# Post-treatment Flare-up Incidence after Using Nano Zinc Oxide Eugenol Sealer in Mandibular First Molars with Irreversible Pulpitis

**DOI:** 10.30476/DENTJODS.2020.83231.1041

**Published:** 2020-12

**Authors:** Maryam Javidi, Mina Zarei, Ehsan Ashrafpour, Maryam Gharechahi, Hossein Bagheri

**Affiliations:** 1 Dept. of Endodontics, Dental Research Center, Faculty of Dentistry, Mashhad University of Medical Sciences, Mashhad, Iran; 2 Postgraduate Dept. of Endodontics, Dental Research Center, Faculty of Dentistry, Mashhad University of Medical Sciences, Mashhad, Iran; 3 Dept. of Dental Material, Dental Research Center, Faculty of Dentistry, Mashhad University of Medical Sciences, Mashhad, Iran

**Keywords:** Flare-up, Nano sealer, AH-26 Sealer

## Abstract

**Statement of the Problem:**

Some patients may report moderate-to-severe pain and/or swelling following root canal treatment, which is undesirable for both the patient and dentist and may require an unscheduled emergency visit by patients to relieve their symptoms.

**Purpose:**

The aim of this study was to evaluate the post-treatment flare-up incidence following application of nano zinc oxide-eugenol (NZOE) sealer in mandibular first molars with irreversible pulpitis.

**Materials and Method:**

This single-blinded study was performed on 60 patients having mandibular first molars with irreversible pulpitis. After signing the written consent form, the patients were randomly divided into two groups considering their age range (20-34 and 35-50 years). Individuals without systemic diseases and with a ﬁrst mandibular molar diagnosed with irreversible pulpitis due to caries, no sinus track and abscess, normal periapical radiographic appearance, no spontaneous pre-treatment pain, not taken any medication for at least 8 hours before the treatment visit were included in this study. Patients of both sexes with age range of 20 to 50 years were selected. In order to obturate the root canal space, AH26 sealer was used in the one group and the synthesized NZOE was applied in another group. The patients were then given questionnaires to report the severity of pain and presence of swelling during 6, 18, 24, and 48 hours of follow-up. The data was analyzed using SPSS v.19 software applying repeated measures ANOVA. The significance level was set at 0.05.

**Results:**

The severity of pain was significantly lower in NZOE group at 24 hours post-treatment (*p*= 0.003). Patients reported no symptoms of swelling in any group.

**Conclusion:**

NZOE sealer manifested satisfactory results and could be regarded as a promising substitute to routine sealers since patients may experience less pain during the first hours after receiving root canal therapy.

## Introduction

The main objectives of root canal therapy are to achieve long-term comfort, function, and aesthetics for the patients and prevention of reinfection of tooth. These objectives are provided through complete cleaning, shaping, and obturation of canals of affected teeth [ 1 ]. However, some patients may report moderate-to-severe pain and/or swelling following root canal treatment that is detrimental for both patient and dentist and may entail an unscheduled emergency visit by patients to relieve their symptoms. The rate of flare-up following root canal therapy (RCT) has been reported to range from 2.53% to 58% [ 2 ]. Pack *et al*. reported 40% of the patients had post-operative pain 24 hours after RCT and this decreased to 11% after one week [ 3 ]. Although the exact reasons for flare-up are not clearly understood, it is accepted that flare-up is a multi-factorial phenomenon and there is no single reason for its occurrence [ 4 ]. Microbiological, chemical, mechanical, and host variables can induce pain and swelling following root canal treatment [ 5 ]. In addition, history of preoperative pain, gender and age factors, the pre-operative pulp status, the type of tooth, the type of treatment (initial or re-treatment), and a history of allergy may all influence the rate of flare-up [ 6 ]. In addition, some studies have demonstrated the effect of several factors such as instrumentation technique, type of analgesic, method of analgesic prescription, type of rotary instrument, anesthetic solution, occlusal reduction, and method of root canal filing on incidence of flare up [ 7 - 8 ]. 

In obturation of the root canal system, sealer is used to prevent penetration of microorganisms and their byproducts. In the other hand, sealer is in direct contact with periapical tissues and may cause inflammation, tissue degeneration, and delay in wound healing. Therefore, the ideal root canal sealer should be non-cytotoxic, non-mutagenic, and immunologically compatible with periapical tissues [ 9 ]. Currently, a large variety of sealers with different formulas and physical properties are available for use. However, they all have their limitations. It is difficult to produce a sealer with proper physicochemical properties while being biocompatible for long-term. For many years, zinc oxide-eugenol (ZOE)-based sealers have been widely used in endodontic practice. The use of nanotechnology has allowed many developments in dentistry and advances in oral-health-related nano material and therapeutic methods [ 10 ]. Some of the advantages of using nano particles in endodontic sealers include improving their physicochemical characteristics, enhancing the antibacterial property, better penetration into the dental tubules, decreasing microleakage, and increasing biocompatibility [ 11 - 12 ]. It has been shown that incorporating zinc oxide nano particles enhances the physicochemical characteristics (setting time, flow, solubility, dimensional stability, and radiopacity) of Grossman sealer [ 12 ]. Kesler Shvero *et al*. [ 13 ] demonstrated that epoxy resin-based surfaces with cationic nano particles attracted and sacrificed Enterococcus faecalis. DaSilva *et al*. [ 14 ] showed that incorporating chitosan nano particles into ZOE sealer reduced the formation of biofilm within the sealer-dentin interface. In addition, it has been reported that nano-ceramic sealer had better cytocompatibility compared to Endoseal MTA considering the effects on cell spreading and proliferation [ 15 ].

Recently, a new endodontic sealer with nano-sized ZO powder particles (NZOE) has been developed in the Dental Material Research Center, Mashhad University of Medical Sciences, Mashhad, Iran. This sealer is similar to various ZOE-based sealers, but with different sizes of ZOE nanoparticles. In previously published articles, we verified that NZOE sealer had less microleakage and better antibacterial property in comparison with Pulpdent and AH-26 sealers [ 16 ]. In an animal study, it was observed that the histocompatibility properties of NZOE were comparable to the above-mentioned commercial sealers [ 17 ]. Moreover, the cytotoxicity of NZOE on murine L929 cell line was comparable to that of Pulpdent and was lower than AH-26 sealer [ 18 ]. In another study, the cytotoxicity of NZOE sealer on human gingival fibroblasts isolated from healthy subjects was evaluated and NZOE exhibited lower cytotoxicity compared to Pulpdent and AH-26 [ 19 ]. 

ZOE -based sealers are the oldest sealer used in endodontic therapy. Zinc oxide (ZO) is a valuable component of these sealers that is very effective as an antimicrobial agent. Many reforms have been done on these sealers in order to improve their properties and many commercial models are available [ 20 ]. In addition, ZOE-based cements have been found to possess favorable characteristics in terms of biocompatibility [ 19 - 20 ].

To the best of our knowledge, the use of nano-structured materials as sealers in root canal therapy is limited to two or three types of nano-structured hydroxyapatite alone or in combination with epoxy resin (Nanoseal) [ 20 - 22 ].

No evidence-based investigations have evaluated postoperative pain and swelling when different sealers are used for obturation. The aim of this study was to assess postoperative pain and swelling in a prospective randomized clinical trial following the use of AH-26 and NZOE as a sealer in mandibular molars with irreversible pulpitis that were treated in one visit.

## Materials and Method

The Ethics Committee of Mashhad University of Medical Sciences (Ir.mums.dentistry.rec.1397.044 and Iranian Registry of Clinical Trials ID No. IRCT2018071136542) approved the protocol of this study.

### Synthesis of ZnO nano-particles

In this work, ZnO nano-powders were prepared by a modified sol-gel method, using gelatin [ 23 ].

To prepare 5 g of the final product, first a solution of gelatin (type B from bovine skin, Sigma Aldrich) was prepared by dissolving 10 g of gelatin in 150 mL of deionized water at 60°C. Then, appropriate amounts of zinc nitrate (Zn (NO3)2 .6H2O; Merck %99) were dissolved in minimum deionized water at room temperature. Then, the two solutions were mixed and stirred for 8 hours while the temperature was kept at 80°C. Finally, the pure resins were calcined at different temperatures of 500, 600, and 700°C to obtain ZnO nano-powders. Morphological and structural properties of the prepared ZnO were characterized by x-ray diffraction (XRD) and transmission electron microscopy (TEM) techniques.

### Procedural steps

The sample size was calculated based on an alpha of 0.05 with power of 80%, indicating that 27 patients would be required in each group but after considering the dropout rate of 10%, the sample size increase to 30 patients in each group. 

Inclusion criteria were as follows: individuals without systemic diseases and with a ﬁrst mandibular molar diagnosed with irreversible pulpitis due to caries, no sinus track and abscess, normal periapical radiographic appearance, no spontaneous pre-treatment pain, not having taken any medication for at least 8 h before the treatment visit. Patients of both genders ranging from 20 to 50 years of age were selected. During the root canal procedure, teeth that could not be treated in a single visit or patients who interrupted treatment were excluded. In addition, the patient with severe periodontal disease, over instrumentation or overﬁlling beyond the root canal space, teeth that could not be isolated with rubber dam, root canal calciﬁcation, root resorption and teeth that were not suitable for further restoration were excluded. 

All the treatment procedures were performed by a postgraduate student in the Endodontic Department, Mashhad Dental School, Iran, from May to August 2018. A total of 60 teeth were randomly assigned to two groups using a random-digit table. Before initiating treatment, the patients were asked to rate their pre-treatment pain on a visual analogue scale from 0 to 9. For each number, one of the sealers was selected. Each patient chose one of the numbers, and based on the number, the nurse supplied one of the sealers for the treatment visit. Since two sealers were different in their shape and appearance, the practitioner could not be blind. Prior to beginning of the root canal treatment, inferior alveolar nerve block injection was done with 2% lidocaine with 1: 80 000 epinephrine. After isolation with a rubber dam, followed by access cavity preparation, working length was determined with a Raypex6 apex locator (VDW, Munich; Germany) and then it was conﬁrmed radiographically. After preparing the root canals with hand instruments to at least a size 15 K-ﬁle (Mani, Togichi; Japan), Hero rotary instruments (Micro-Mega, Besançon; France) were used for root canal preparation. Apical patency was performed with a size 10 K-ﬁle between each rotary instrument. The apical preparation was completed up to size 30, .04 taper. Between instruments, the root canals were irrigated with 3 mL of 5.25% NaOCl with a 30-gauge side perforated needle (Endo-Top, PPH Cerkamed, Stalowa Wola; Poland). During irrigation, the needle was repeatedly moved up and down to prevent locking in the canals. The smear layer was removed by irrigating with 3 mL 17% Ethylene diamine tetra acetic acid (EDTA) (Asia Chemi Teb Co., Tehran, Iran) followed by 5 mL normal saline irrigation at the end of root canal preparation. The root canals were dried with paper points (Meta Biomed Co., Chungcheongbuk; Korea). Then in the group 1, the canals were ﬁlled with gutta-percha (Meta Biomed Co.) and AH26 (Dentsply DeTrey, Konstanz; Germany) sealer and group 2 was filled with gutta-percha and NZOE sealer using the cold lateral condensation technique. Each patient was given a numerical visual analogue scale (VAS) form to record the severity of pain from 0 to 9 during the 6, 18, 24, and 48-hours period following treatment, and the other form was used to record swelling. In the second form, the following scores were used: 0 for none swelling, and 1 for swelling. The data were analyzed using SPSS v.19 software applying Chi-square and Kendall’s Tau-b. The significance level was set at 0.05.

## Results

Based on the inclusion and exclusion criteria, a total of 64 patients were eligible to participate in the study. Four patients from both groups were excluded from study since treatment could not be completed in one visit for one patient; two patients did not return the VAS form, and one patient had partial necrosis pulp. Finally, 60 patients were included for data analysis (30 in each group. The age and sex distribution of patient in each group was reported in Table 1. The two groups were similar according to distribution of age and gender of the patients. None of the patients had pain on palpation, percussion and spontaneous pain at the treatment visit. The results showed that at the 24 h following treatment, the patients receiving NZOE as the sealer had experienced statistically signiﬁcant lower pain compared to those who had AH 26 (p= 0.003); no signiﬁcant difference was found between the group for the rest of the study defined time periods (Table 2). None of the patient in both groups had swelling during the 48 h period following treatment.

**Table 1:** Frequency distribution of gender and age in sealer groups

Variable		AH-26	NZOE	Total	*p* Value
		N(%)	N(%)	N(%)	
Age	20-34 years	16(53.3)	16(53.3)	32(53.3)	1.00
	35-50 years	14(46.7)	14(46.7)	28(46.7)	
Sex	Male	15(50)	14(53.3)	29(48.3)	0.796
	Female	15(50)	16(46.7)	31(51.7)	

N:Number

**Table 2:** Effect of AH 26 and NZOE on pain in different periods

Time	Sealer	No pain	Very low	Low	Moderate	Severe	Very severe	*p* Value
		n(%)	n(%)	n(%)	n(%)	n(%)	n(%)	
6h	AH26	0(0.0)	0(0.0)	0(0.0)	8(44.4)	10(55.6)	0(0.0)	0.872
	NZOE	0(0.0)	0(0.0)	0(0.0)	0(0.0)	5(41.7)	7(58.3)	
18h	AH26	0(0.0)	1(5.6)	7(38.9)	10(55.6)	0(0.0)	0(0.0)	0.107
	NZOE	1(8.3)	5(41.7)	1(8.3)	4(33.3)	1(8.3)	0(0.0)	
24h	AH26	0(0.0)	5(27.8)	13(72.2)	0(0.0)	0(0.0)	0(0.0)	0.003
	NZOE	0(0.0)	7(58.3)	5(41.7)	0(0.0)	0(0.0)	0(0.0)	
48h	AH26	11(61.1)	7(38.9)	0(0.0)	0(0.0)	0(0.0)	0(0.0)	0.119
	NZOE	3(25.0)	9(75.0)	0(0.0)	0(0.0)	0(0.0)	0(0.0)	

## Discussion

In the present study, the results of single-visit root canal treatment of mandibular first molars with irreversible pulpitis with the use of AH26 and NZOE as root canal sealers showed that the NZOE was associated with statistically signiﬁcant less pain at the 24 h after treatment. Moreover, postoperative pain intensity decreased with time within both groups that was in agreement with the findings of Parirokh *et al*. [ 8 ]. In both experimental groups, no swelling was found during the 48 h follow up period of study. This study was the first clinical study performed on this new sealer.

In the present study, for prevention of possible bias, patients with similar preoperative conditions were included. As the preoperative pain would have a signiﬁcant effect on postoperative pain [ 24 ], only patients with no spontaneous pain were included. In addition, because pre-treatment analgesic consumption would influence the postoperative pain following root canal treatment [ 25 ], all patients were asked about the medication they used. The participants should not have taken any medication for at least 8 h before the treatment visit. According to systematic reviews, single-visit root canal treatment has no signiﬁcant difference with multi-visit treatment regarding postoperative pain [ 26 ]. Therefore, in the present study, single-visit root canal treatment was undertaken. In this study, the distribution of age and gender were the same for both groups due to the established association of these variables to endodontic flare-up. All the treatments carried on mandibular first molars with irreversible pulpitis and instrumentation of the root canals was completed in a crown-down technique using rotary nickel titanium files as it has shown to be associated with less debris and irrigants extrusion apically, which has a clinical implication in decreasing the incidence of postoperative pain [ 27 ]. In addition, attempt was done to avoid over-instrumentation of root canal during filing. Therefore, both experimental groups were identical in terms of age, gender, tooth type, preoperative pain intensity, irrigation solution, instrumentation, and obturation technique. The results of a systematic review showed that during the first 48 h after root canal treatment, post- operative pain decreased signiﬁcantly [ 28 ]. Therefore, we limited our research to the ﬁrst 48 h after treatment. 

Various methods were used for evaluating the effect of different variables on postoperative endodontic pain. In the present study, a VAS was used to compare postoperative pain intensity because it is a simple tool with high reliability and validity and more sensitive to small changes than descriptive ordinal scales [ 8 ].

There are some studies on the effect of irrigation type [ 29 ] and irrigation concentrations [ 30 ] on endodontic flare up but no study was evaluated the relationship of endodontic sealers and flare up. In the other hand, some researchers evaluated the effects of sealer extrusion into the periapical tissues. Sometimes, extrusion of sealers or diffusion of toxic components from the sealers may occur into the surrounding anatomical structures and periapical tissues during root canal obturation. Although small material extrusions are generally well tolerated by the periradicular tissues and have no significant effect on periapical healing and endodontic outcome, clinical symptoms such as pain and swelling may appear. Toxic effects of the products and a pressure phenomenon from the presence of sealer can initiate an inflammatory process and tissue damage and lead to flare up [ 31 ].

Over-instrumentation, excessive amount of sealer, the excessive compaction force, hydrostatic pressure, the use of lentulo spirals, immature canal apices, and root tip resorption can increase the risk of sealer extrusion [ 32 ].

In this study, the most post-operative pain was reported by AH 26 group during the 24 h after treatment. This can be due to mild-to-moderate irritating effects of AH26 when freshly prepared and the toxicity is due to the release of a small amount of formaldehyde, epoxide bisphenol resin, or amines during the chemical setting process [ 33 ]. In addition, Ruparel *et al*. [ 34 ] revealed that resin-based sealer can directly activate trigeminal nociceptors and leading to release of CGRP and may therefore cause pain and neurogenic inflammation. In another study, Huang *et al*. [ 35 ] reported that AH26 sealer had significant cytotoxicity in the periradicular tissues by inducing inflammatory mediators such as receptor activator of nuclear factor kappa B ligand, nitric oxide synthase, cyclooxygenase- 2, and reactive oxygen species. The direct activation along with the immunologic response may underlie flare-up occurrences. On the other hand, two in vitro studies reported that the cytotoxicity of NZOE on murine L929 cell line [ 18 ] and on human gingival fibroblasts [ 19 ] was lower than AH-26 sealer. 

According to the result of the present study and considering the lesser microleakage and better antibacterial property of NZOE in comparison to AH-26 sealers [ 16 ] and concerning the lower cytotoxicity of NZOE than AH-26 sealer [ 17 - 19 ], NZOE sealer could be regarded as endodontic sealer for root canal obturation.

## Conclusion

Up to 24 h following root canal treatment, the use of NZOE sealer was associated with signiﬁcantly lesser pain than the use of AH26 in studied patients. 
